# Olaparib significantly delays photoreceptor loss in a model for hereditary retinal degeneration

**DOI:** 10.1038/srep39537

**Published:** 2016-12-22

**Authors:** Ayse Sahaboglu, Melanie Barth, Enver Secer, Eva M. del Amo, Arto Urtti, Yvan Arsenijevic, Eberhart Zrenner, François Paquet-Durand

**Affiliations:** 1 Institute for Ophthalmic Research, Tuebingen, Germany; 2 Graduate Training Center of Neuroscience, Tuebingen, Germany; 3 Department of Medical Genetics, Erciyes University, Kayseri, Turkey; 4 School of Pharmacy, University of Eastern Finland, Kuopio, Finland; 5 Centre for Drug Research, Division of Pharmaceutical Biosciences, University of Helsinki, Helsinki, Finland; 6 Hôpital Ophtalmique Jules Gonin, Lausanne, Switzerland

## Abstract

The enzyme poly-ADP-ribose-polymerase (PARP) mediates DNA-repair and rearrangements of the nuclear chromatin. Generally, PARP activity is thought to promote cell survival and in recent years a number of PARP inhibitors have been clinically developed for cancer treatment. Paradoxically, PARP activity is also connected to many diseases including the untreatable blinding disease Retinitis Pigmentosa (RP), where PARP activity appears to drive the pathogenesis of photoreceptor loss. We tested the efficacy of three different PARP inhibitors to prevent photoreceptor loss in the *rd1* mouse model for RP. In retinal explant cultures *in vitro*, olaparib had strong and long-lasting photoreceptor neuroprotective capacities. We demonstrated target engagement by showing that olaparib reduced photoreceptor accumulation of poly-ADP-ribosylated proteins. Remarkably, olaparib also reduced accumulation of cyclic-guanosine-monophosphate (cGMP), a characteristic marker for photoreceptor degeneration. Moreover, intravitreal injection of olaparib in *rd1* animals diminished PARP activity and increased photoreceptor survival, confirming *in vivo* neuroprotection. This study affirms the role of PARP in inherited retinal degeneration and for the first time shows that a clinically approved PARP inhibitor can prevent photoreceptor degeneration in an RP model. The wealth of human clinical data available for olaparib highlights its strong potential for a rapid clinical translation into a novel RP treatment.

The enzyme poly(ADP-ribose) polymerase (PARP) is one of the key mediators of DNA damage repair^1^ and generally seen as a beneficial factor in cell physiology. However, PARP activity is also connected to a variety of human diseases, essentially in two different ways: 1) in cancer, the repair of DNA damage allows cells to survive and possibly contributes to cancerogenesis; 2) in neurodegenerative diseases, excessive activation of PARP may deplete cellular substrates and lead to a specific form of programmed cell death, termed PARthanatos^2^. Thus, PARP seems to be localized at a cross-road of cell physiology and pathology. The tight control of its activity is a major focus in recent therapy developments.

Retinitis pigmentosa (RP) is a group of hereditary retinal degenerative diseases in which rod photoreceptors die due to a genetic mutation, whereas cone photoreceptors disappear secondarily, once rods are gone. While the initial disease symptoms (*i.e.* night blindness) are comparatively mild, the secondary loss of cones ultimately leads to complete blindness. The disease affects approximately 1 in 3,000 to 7,000 people^3^ and is characterized by strong genetic heterogeneity with causative mutations in more than 65 genes. In 4–8% of human RP cases, the disease is caused by mutations in the genes encoding for cGMP specific phosphodiesterase 6 (PDE6)^4^,^5^. The non-functional enzyme fails to hydrolyze cGMP, causing its accumulation^4^,^6^. Animal models like the retinal degeneration 1 (*rd1*) mouse, which harbors a mutated *Pde6b* gene^7^, have advanced the understanding of the cellular processes underlying retinal degeneration. Notably, elevated cGMP levels in dying photoreceptors were found to correlate with increased activity of PARP^8^,^9^.

PARP is an important mediator of base excision repair. It has three zinc finger domains that differentially recognize DNA double strand breaks and single strand breaks^10^. DNA damage activates PARP to catalyze extensive polymerization of ADP-ribose from NAD^+^ onto acceptor proteins, for instance histones and PARP itself^11^. The cofactor of PARP is nicotinamide adenine dinucleotide (NAD) and sustained PARP activity following excessive DNA damage decreases NAD^+^ levels in a dose-dependent manner^12^. Consequently, ATP levels will fall because NAD^+^ is needed for glycolysis and the Krebs cycle^13^. Berger proposed a mechanism, known as the “PARP suicide hypothesis”, suggesting that excessive activation of PARP may account for rapid cell death before DNA repair can take place^14^. This kind of cell death, later named ‘parthanatos’ (derived from the Greek Θάνατος, “Death”) is associated with nuclear translocation of the mitochondrial protein apoptosis-inducing factor (AIF)^15^ and energy depletion^16^. Although NAD^+^ and ATP depletion appear to be relatively early events after PARP activation, cell death only takes place many hours later^17^, indicating that other downstream mediators may be present and epigenetic changes, *e.g.* cytosine methylation, are involved. This corresponds to similar observations in *rd1* photoreceptors, both in terms of cell death timing^18^ and in dramatically altered gene expression found in *rd1* retinas^19^. Moreover, the methylated and hydroxymethylated form of cytosine (5mC and 5hmC) accumulate in *rd1* retinas^20^,^21^, implying dynamic changes in global epigenetic regulation during retinal degeneration.

The retina of mice in which PARP-1 was genetically deleted is morphologically and functionally normal, but resistant to PDE6 inhibition-induced retinal degeneration^9^, suggesting that PARP-1 in particular is responsible for photoreceptor degeneration. In a comparative study, excessive PARP activity was found to be a common denominator for photoreceptor cell death in ten different retinal degeneration models, including in the *rd1* mouse^22^ highlighting the potential of PARP inhibitors for the treatment of genetically diverse groups of RP patients.

Here, we tested three recently developed PARP inhibitors for photoreceptor neuroprotective capacities. Among the inhibitors tested, the phthalazinone-based olaparib, an FDA approved drug for the treatment of ovarian cancer^23^ markedly reduced photoreceptor degeneration *in vitro* and *in vivo*. Our data confirms the importance of PARP activity for photoreceptor degeneration and suggests olaparib for a rapid clinical translation into a treatment for RP.

## Results

Previously, we had found that the 1^st^ generation PARP inhibitor PJ-34 afforded moderate but significant photoreceptor protection in *rd1* retina^8^. Recently, several PARP inhibitors have been developed clinically and we decided to test three promising compounds for their photoreceptor protective capacities, initially in organotypic retinal explant cultures derived from *rd1* animals. The PARP inhibitors tested were: R503, an experimental compound developed by the company Radikal Therapeutics; ABT-888 (Veliparib), a PARP inhibitor currently being used in several phase III clinical trials (NCT02264990, NCT02163694, NCT02152982); and olaparib (Lynparza^TM^), a drug approved in 2014 for the treatment of ovarian cancer positive for BRCA1/2 mutations. *rd1* retinal explants cultured from post-natal (P) day 7 to 11 with either ABT-888 or R503 exhibited clear signs of toxicity at concentrations of 0.1 μM or 1 μM, respectively (Supplementary Fig. 1). We also observed a disruption of the normal retinal layering with both ABT-888 and R503, suggesting adverse effects on early post-natal retinal development. However, in this initial drug screening olaparib, a drug targeting in particular PARP-1 and PARP-2^24^, appeared to show strong photoreceptor protective effects, calling for a more thorough evaluation of this compound.

### PARP inhibition with olaparib rescues photoreceptor cell death in *rd1* retinal explant cultures

**Figure 1:**
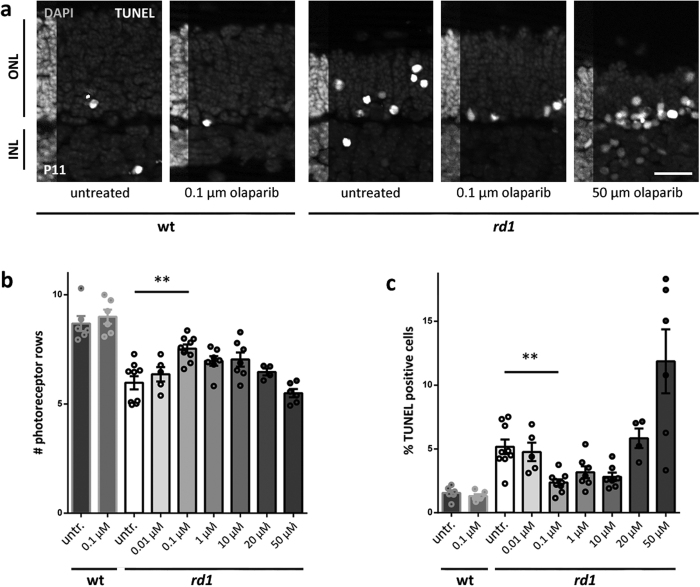
Olaparib rescues *rd1* photoreceptors in short-term retinal explant cultures. (**a**) Immunohistochemical staining revealed a dose-dependent effect of olaparib treatment with 100 nM as the most protective concentration and toxic effects of high concentrations. (**b**) Quantification of photoreceptor rows. (**c**) Quantification of the percentage of TUNEL positive cells. Bar graphs represent means ± SEM. n(wt, untreated) = 6; n(wt, 0.1 μM olaparib) = 6; n(*rd1*, untreated) = 9; n(*rd1*, 0.01 μM olaparib) = 5; n(*rd1*, 0.1 μM olaparib) = 9; n(*rd1*, 1 μM olaparib) = 7; n(*rd1*, 10 μM olaparib) = 7; n(*rd1*, 20 μM olaparib) = 4; n(*rd1*, 50 μM olaparib) = 6. **p < 0.01 by Kruskal-Wallis test for multiple analysis; scale bar is 20 μm.

The effect of olaparib was assessed by counting both surviving photoreceptor rows and dying TUNEL positive cells. Olaparib appeared to have a dose-dependent protective effect on retinal explant cultures with a maximum preservation of photoreceptor rows and a minimum number of TUNEL positive cells at a concentration of 100 nM olaparib. In wildtype (wt) cultures the number of photoreceptor rows and the percentage of TUNEL positive cells in 100 nM olaparib treated *rd1* cultures approached the level of untreated wt, with no other adverse effects seen (Fig. 1). We used DMSO as a solvent for olaparib and since a recent study found toxic effects of DMSO in the retina^25^, we examined whether the DMSO concentrations used in control explants (0.6–30 μM) influenced photoreceptor survival. When the DMSO concentration of control groups for each experiment was calculated and plotted against the average number of photoreceptor rows and the percentages of TUNEL positive cells, no disturbances due to DMSO were found, indicating that the solvent had not influenced the course of degeneration (Supplementary Fig. 2).

### Olaparib decreases PARylation and cGMP levels in rd1 retinal explant cultures

**Figure 2:**
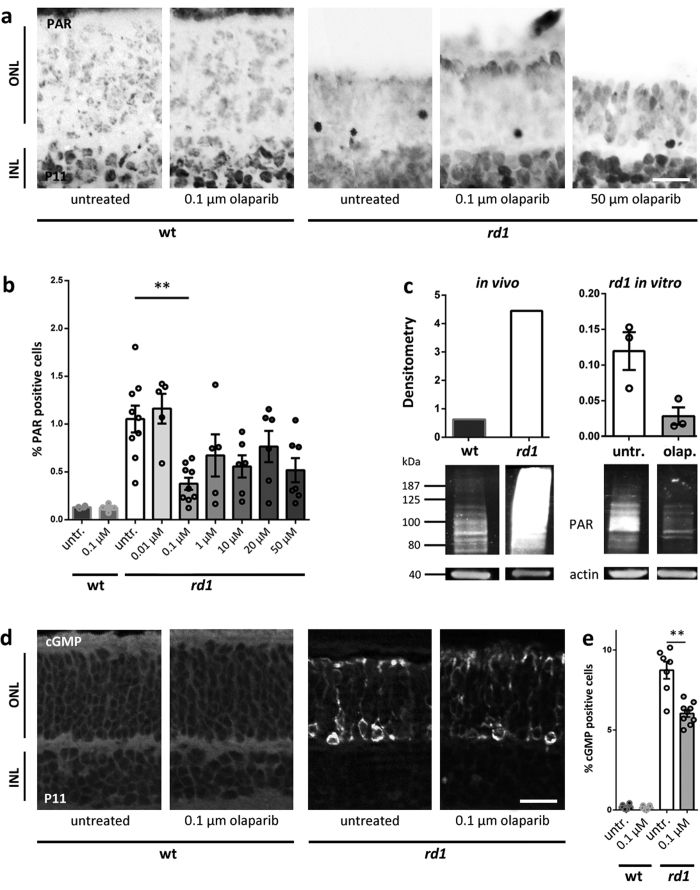
Effect of PARP inhibition with olaparib on PARylation and cGMP levels in *rd1* retinal cultures. (**a,b**) The higher the concentration of olaparib, the smaller was the percentage of PAR positive cells. (**c**) Western blot analysis confirmed the previously reported strong PARylation difference between *rd1* and wildtype (wt) retinas^8^,^9^. Furthermore, there was a strong reduction of PARylation in *rd1* cultures treated with 100 nM olaparib compared to control cultures. (**d**) Immunohistochemical stainings showed an increase of cGMP level in *rd1* compared to wt. (**e**) Quantification of the cGMP signal revealed a significant reduction due to treatment with olaparib. Bar graphs represent means ± SEM. n(wt, untreated) = 6; n(wt, 0.1 μM olaparib) = 6; n(*rd1*, untreated) = 9; n(*rd1*, 0.01 μM olaparib) = 5; n(*rd1*, 0.1 μM olaparib) = 9; n(*rd1*, 1 μM olaparib) = 7; n(*rd1*, 10 μM olaparib) = 7; n(*rd1*, 20 μM olaparib) = 4; n(*rd1*, 50 μM olaparib) = 6; n(WB, *in vivo, rd1*) = 1; n(WB, *in vivo*, wt) = 1; n(WB, *in vitro, rd1*, untreated) = 3; n(WB, *in vitro, rd1*, olaparib) = 3. **p < 0.01 by Kruskal-Wallis test for multiple analysis; scale bars in (**a** and **d**) are 20 μm.

The efficacy of PARP inhibition was assessed using an immunostaining for PAR residues in individual photoreceptor cells. There was a significant decrease in the numbers of photoreceptors showing PAR accumulation in 100 nM olaparib treated *rd1* retinal cultures, while 100 nM olaparib did not affect the numbers of PAR positive cells in wt cultures (Fig. 2a,b). Remarkably, higher concentrations of olaparib did not further reduce the PAR signal, indicating that PARP isoforms other than PARP-1 or PARP-2 may also have contributed to the total PAR accumulation found in *rd1* photoreceptors. Western blot analysis in principle confirmed the immunohistochemistry results. For this, *rd1* and wt *in vivo* retina were used as positive and negative controls, respectively, showing a strong increase in PARylated proteins in *rd1* retina *in vivo,* in line with earlier publications^8^,^9^. Cultured, *in vitro* retina showed an overall lower level of protein PARylation than *in vivo* samples, together with a numerical reduction of PARylation in *rd1* retinal explants treated with olaparib *in vitro* (Fig. 2c; Fig. S5). However, since the number of cells showing strong PARylation at any given time-point is relatively low (approx. 1% of ONL cells; >0.5% of all cells in the retina), the western blot analysis at the whole tissue level failed to show a statistically significant effect. Therefore, for all later analysis, we focused on methods allowing for cellular resolution (*i.e.* TUNEL assay, PAR immunostaining).

Previous studies indicated that increased cGMP levels due to PDE6 dysfunction are reduced in PARP-1 knockout (KO) retina^9^. Thus, the effect of pharmacological PARP inhibition on cGMP levels was assessed and, remarkably, the strong increase of cGMP levels in *rd1* was significantly reduced upon olaparib treatment (Fig. 2d,e).

### Olaparib shows sustained protective effects

**Figure 3:**
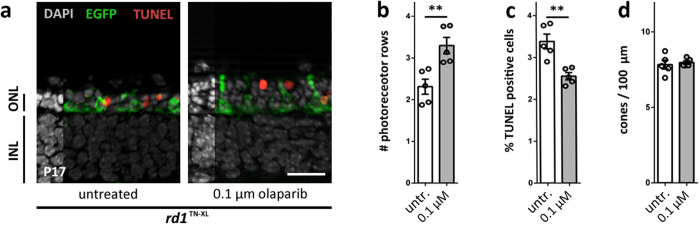
Long-term protective effects of olaparib treatment in *rd1*^TN-XL^ retinal cultures at P17. (**a**) Immunohistochemical staining, at P17, showed improved photoreceptor viability in 0.1 μM olaparib treated cultures, when compared to untreated (untr.) control. (**b**) The number of photoreceptor rows was significantly increased after treatment. (**c**) There were less TUNEL positive cells in olaparib-treated cultures. (**d**) Cone density (EGFP signal) remained unchanged after treatment. Bar graphs represent means ± SEM. n(*rd1*, untreated) = 5; n(*rd1*, 0.1 μM olaparib) = 5. **p < 0.01 by two-sample Komolgorov-Smirnov test; scale bar is 20 μm.

To evaluate the long-term effects of olaparib on *rd1* retinal explant cultures, the treatment paradigm was extended to P17. Since cone photoreceptors are fully differentiated at this age, for these experiments, we used *rd1* mice carrying the TN-XL biosensor^26^ (*i.e. rd1*^TN-XL^) to directly visualize cone survival. Photoreceptor rows were increased after treatment (Fig. 3a,b), whereas the percentage of TUNEL positive cells in olaparib treated cultures was decreased (Fig. 3a,c). Cone density, on the other hand, was unaffected (Fig. 3a,d). Finally, the *in vitro* treatment was prolonged even further to P24. However, here, olaparib had no significant effect on photoreceptor rows, TUNEL positive cells, and cone density (Supplementary Fig. 3), indicating that the treatment delayed photoreceptor degeneration, but could not entirely prevent it in the long-term.

### The effect of PARP inhibition on DNA hypermethylation

**Figure 4:**
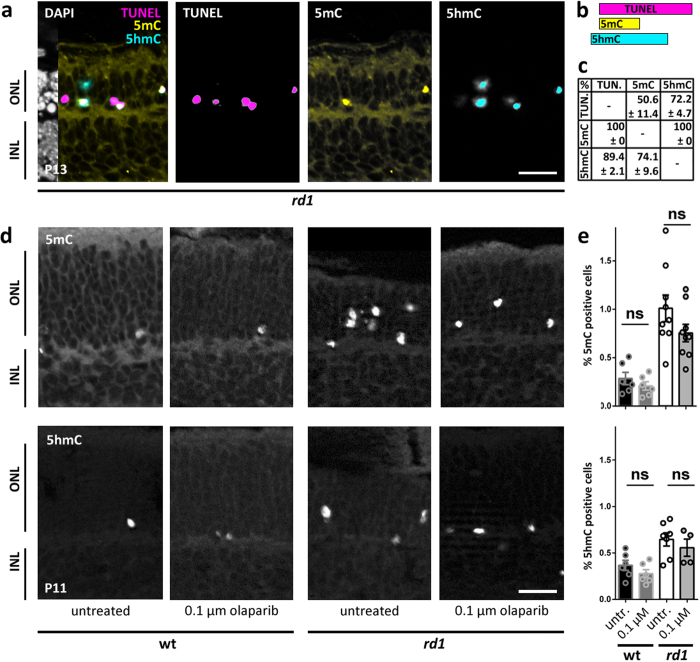
Olaparib does not affect increased DNA methylation. (**a**) Co-localization of TUNEL, 5mC, and 5hmC on *rd1* retina at P13. (**b**) Of the TUNEL positive cells (n = 69), about half were also 5mC positive and nearly all cells were 5hmC positive. All 5mC positive cells (n = 29) were also TUNEL positive, whereas of all 5hmC positive cells (n = 57), there was a small fraction (n = 6) that did not show TUNEL signal. (**c**) Quantification of co-localization, in rows, *e.g.* of all TUNEL positive cells 50.6 ± 11.4 were 5mC and 72.2 ± 4.7 were 5hmC positive. (**d**) Immunohistochemical staining showed an increase of both 5mC and 5hmC levels in *rd1* retinal cultures compared to wildtype (wt). (**e**) Quantification of histological methylation signal. In *rd1* cultures there was no significant reduction after treatment with olaparib (5mC: p = 0.3517; 5hmC: p = 0.7858). Bar graphs represent means ± SEM. n(*rd1* P13) = 4; n(wt, untreated) = 6; n(wt, olaparib) = 6; n(*rd1*, untreated) = 10; n(*rd1,* olaparib) = 8. Tested by two-sample Komolgorov-Smirnov test. Scale bars in (**a** and **d**) are 20 μm.

Epigenetic changes are often reflected in alterations of the methylation level of cytosine in the DNA. Indeed, DNA methylation was recently found to be strongly increased in dying photoreceptor cells^20^,^21^ and this is correlated to a strong over-activation of PARP. To investigate whether DNA methylation and PARP activity were causally connected to each other, we examined global DNA methylation in the ONL of both wt and *rd1* retinal explant cultures, by staining for 5-methyl-cytosine (5mC) and 5-hydroxy-methyl-cytosine (5hmC). At P13, at the peak of degeneration in *rd1* retinas *in vivo*, high levels of 5mC co-localized completely with the TUNEL assay. 5hmC positive cells on the other hand showed a 90% overlap with the TUNEL signal (Fig. 4a–c). Similarly, in retinal cultures at P11, *rd1* retinal explants showed a heavy increase in 5mC and 5hmC DNA methylation of photoreceptors compared to wt. However, inhibition of PARP with 100 nM olaparib did not significantly decrease the levels of 5mC or 5hmC (Fig. 4d,e), indicating that DNA methylation was either unrelated to or upstream of PARP activity.

### Olaparib protects rd1 photoreceptors *in vivo*

The *in vitro* data suggested olaparib as a promising compound for *in vivo* application in the *rd1* mouse, with an effective dose to lie between 0.01–0.1 μM. Although olaparib is known to be well tolerated when given systemically^23^, we wanted to avoid the possibility of any systemic side-effects and therefore decided to use direct application to the eye via intravitreal injection. To guide and optimize the *in vivo* paradigm, we used the recently developed Quantitative Structure-Property Relationships (QSPR) mode^27^ to predict an intravitreal clearance for olaparib of 0.665 ml/h in rabbit eye, while in mouse eye, based on size scaling it is expected to be of 0.021 ml/h. Furthermore, the intravitreal half-life of olaparib (t_1/2_ = ln2 × vitreal volume/clearance) in mouse eye was estimated to be eight minutes. This estimate should be considered a theoretical minimum; the intravitreal half-life may be extended, for instance, if olaparib was bound to specific proteins in the vitreous.

**Figure 5:**
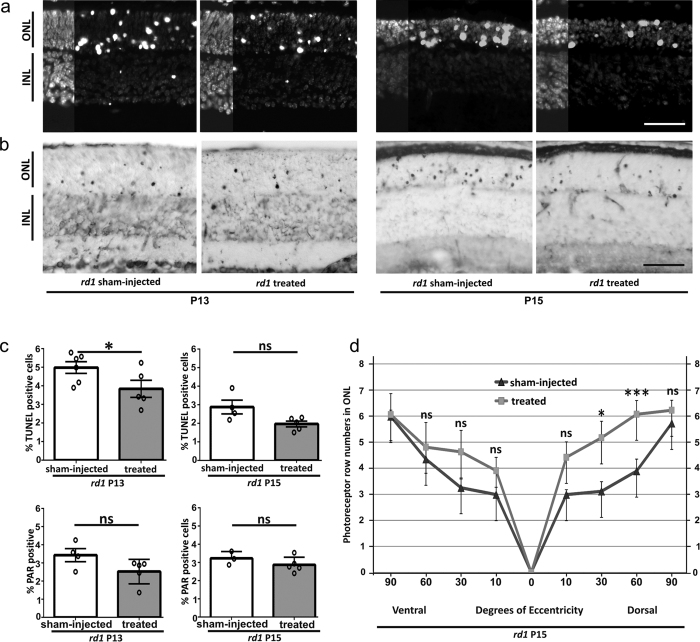
Olaparib protects *rd1* photoreceptors *in vivo*. After a single intravitreal olaparib injection at P11 *rd1* animals where analyzed for cell death (TUNEL assay) and photoreceptor survival at P13 and P15. (**a**) Retinas from sham-injected *rd1* animals display a high number of dying, TUNEL positive cells in the ONL. At P13, in treated animals cell death is reduced, an effect that is still apparent at P15. Note the decrease in the overall size of the *rd1* ONL and the number of photoreceptor rows at P15. (**b**) A subset of *rd1* photoreceptors showed a strong immunoreactivity for PARylated proteins, their numbers appeared lower in P13 and P15 treated animals. (**c**) Top panel: Quantification of TUNEL positive cells in untreated *vs*. treated animals at P13 and P15. At P13 there were significantly less photoreceptors dying in treated *rd1* retina. Bottom panel: Quantification of PAR positive cells in untreated *vs*. treated animals at P13 and P15. (**d**) Quantitative analysis of photoreceptor survival along the dorso-ventral axis. While untreated P15 *rd1* retina displayed 3-6 photoreceptor rows, olaparib treated *rd1* animals had up to two rows more photoreceptors in the mid-periphery, dorsal retina. n(*rd1*, sham-injected P13) = 6; n(*rd1*, treated P13) = 5; n(*rd1*, sham-injected P15) = 4; n(*rd1*, untreated P15) = 5. Tested by unpaired, two-tailed Student’s t-test. Scale bars in (**a** and **b**) are 50 μm.

We then chose a 1 μM olaparib solution for intravitreal injection, giving an effective concentration of 0.1 μM when assuming even intraocular distribution. This allowed being well below the toxic dose while remaining in an effective dose range for at least four half-lifes (*i.e.* at least 30 min). After a single intravitreal injection of 0.1 μM olaparib at P11, the injected eye’s retina showed a strong decrease in the numbers of dying ONL cells, at P13, as assessed with the TUNEL assay, when compared to the sham-injected contralateral eye (Fig. 5a,b). At P15 the olaparib treated eye still displayed a numerical decrease of dying cells in the ONL assessed over the whole retina, but this effect was no longer statistically significant. Similarly, PAR immunohistochemistry showed a decrease of PAR positive cells in the ONL of treated eyes at both P13 and P15; however, this effect did not attain statistical significance. Importantly, when P15 photoreceptor survival was analyzed along the dorso-ventral axis, the spider plot for the numbers of ONL photoreceptor rows showed a statistically significant increase in photoreceptor numbers in the dorsal retina (Fig. 5d).

## Discussion

Excessive activation of PARP has been connected to hereditary photoreceptor degeneration in a large variety of relevant animal models^22^. Here, we show that olaparib, a 3^rd^ generation PARP inhibitor that was recently approved for the treatment of ovarian cancer^23^, rescued mutant photoreceptors both *in vitro* and *in vivo* at nanomolar concentrations. These results highlight olaparib as a candidate drug for the rapid clinical translation into a treatment for currently still untreatable hereditary retinal degeneration.

In many retinal degeneration animal models the causative genetic mutations lead to dysregulated cGMP levels^22^. In *rd1* rod photoreceptors cGMP levels rise because of their non-functional phosphodiesterase 6 (PDE6)^6^,^28^ and this is closely correlated to over-activation of PARP and photoreceptor PAR accumulation^8^,^9^. Interestingly, olaparib inhibition of PARP – which is thought to be downstream of cGMP-signaling – could significantly lower abnormally high cGMP levels. This finding corresponds to a similar observation on reduced photoreceptor cGMP levels in retina obtained from PARP-1 KO mice^9^. Yet, PARP inhibition in isolated coronary arterioles had the exact opposite effect on cGMP: There the PARP inhibitors ABT-888 and INO1001 increased the activity of nitric oxide synthase (NOS) and soluble guanylyl cyclase (sGC) to result in a net increase of cGMP production (Choi *et al*. 2012). However, in photoreceptors neither NOS nor sGC^29^ are expressed so that this pathway to raise cGMP is unavailable. Instead in photoreceptors, PARP activity and cGMP-signaling may be connected in at least two possible ways: (1) via PARP-dependent regulation of gene expression^30^, which could have a bearing on GC or on GC regulating enzymes^31^. (2) via clearance of cGMP, which can be shuttled by ATP-binding cassette family (ABC) pumps to the extracellular space^32^. ATP depletion due to excessive PARP activity would impair the ATP-driven removal of cGMP, whereas PARP inhibition would rescue this effect. The latter possibility might also explain why the effect of PARP inhibition on PAR accumulation (down to approx. one third) is more pronounced than the effect on cGMP accumulation (down to approx. 70%). In the future, it may be interesting to study these negative feedback effects to identify further mediators of the degenerative processes.

Epigenetics likely play an important role in programmed cell death in the retina^33^,^34^. Via alterations of PARP-DNA complexes and corresponding changes in DNA replication and transcription, PARP inhibitors could bring about indirect changes in epigenetic signatures that may be independent of their direct effect on PARP catalytic activity^35^. Furthermore, the activation of PARP may be related to the upstream activity of histone deacetylases (HDAC)^33^,^36^. While both HDAC and PARP influence histone and chromatin structure, epigenetic processes may also target the DNA structure. One repressor mark is 5mC, which is known to recruit proteins that can mediate the activation of co-repressor complexes to target promoters^37^. In *rd1* retinal degeneration, both 5mC and 5hmC were found to be increased and colocalized with TUNEL staining^20^,^21^. 5hmC is generated from 5mC by the activity of ten eleven translocation (Tet), and can be further processed to 5-formylcytosine (5fC) and 5-carboxylcytosine (5caC) by Tet family members. 5hmC appears to function as an active mark at enhancers, and thus 5mC and 5hmC might have reciprocal roles in the dynamic regulation of DNA methylation^37^. Therefore, a balance of 5mC and 5hmC is likely to be important for the homeostasis of postmitotic neurons, where 5hmC is particularly abundant^38^. The loss of photoreceptors in *rd1* retinas has been found to be accompanied by high levels of 5mC and 5hmC and both modifications seem to colocalize with TUNEL and moreover with PAR^20^. This suggests that DNA hypermethylation plays an important role in retinal cell death. While all 5mC positive cells were also TUNEL positive, this was not the case for all of the 5hmC positive cells. This would imply that DNA hypermethylation could follow a specific sequence with the dying cell first turning 5hmC and then 5mC positive. Since olaparib treatment did not reduce 5 mC and 5hmC levels, cytosine methylation is likely to be upstream of excessive PARP activity, or may be the result of a parallel process following dysregulated cGMP levels.

Several previous studies have suggested PARP inhibition as a therapeutic strategy to treat RP^8^,^9^,^22^; however, now it is important to identify PARP inhibitors suitable for long-term use in a chronic human disease. In recent years, numerous PARP inhibitors have undergone clinical trials – mostly for cancer therapy – and a large amount of human tolerability and efficacy data is available today^39^. This in turn should facilitate and accelerate clinical trials and repurposing of PARP inhibitors for RP. While for cancer treatment PARP inhibition aims to disrupt DNA repair so as to cause cell death, in retinal neurodegeneration, pathological over-activation of PARP needs to be prevented. Olaparib is a novel PARP inhibitor with increased specificity for PARP-1 and -2^24^ that was approved for the treatment of ovarian cancer positive for BRCA1/2 mutations (FDA reference ID: 3675412) in 2014. In our retinal explant cultures olaparib showed a significant reduction of PARylation and cell death and, conversely, an increase in photoreceptor survival already at 0.1 μM. Moreover, the therapeutic range was large as only concentrations about 200 fold higher showed toxic effects. While another PARP inhibitor, PJ-34, was found to reduce cell death in the ONL by 20% at the most protective concentration of 6 μM^8^, 0.1 μM olaparib was able to decrease cell death by 50%. Moreover, the protective effects were still visible at P17 *in vitro*, with no obvious detrimental effects until P24. The *in vivo* application of olaparib, however, still faces the problem of sustained delivery to the photoreceptors. While PARP inhibition at P11 caused a transient decrease of TUNEL positive, dying cells at P13, this effect on cell death was no longer significant at P15, even though the numbers of surviving photoreceptors were still higher at this time-point. Thus, in the future it will be important to identify drug delivery vehicles^40^ that will allow for a long-term intravitreal release of olaparib with as few as possible applications.

Here, we show that the clinically approved PARP inhibitor olaparib significantly increases photoreceptor survival in *rd1* retinal explant cultures, in both the short- and long-term. Additionally, PARP inhibition significantly reduced cGMP levels, while it did apparently not affect DNA methylation. Remarkably, a single intravitreal dosing of olaparib significantly preserved *rd1* photoreceptors up to four days post-injection. The importance of these findings lies in the fact that olaparib is already clinically used and regarded as safe when given systemically to patients. This may allow for a rapid clinical translation and the development of olaparib treatment for RP and related neurodegenerative diseases of the retina, either in a local treatment to the eye (*e.g.* via intravitreal injection) or even in systemic treatments.

## Methods

### Animals

C3H *rd1* and wildtype (wt) mice^41^ were used. For cone examination in long-term experiments, HR2.1:TN-XL x *rd1 (rd1*^TN-XL^) animals were used that stably express the TN-XL biosensor in cones^29^. Animals were housed under standard white cyclic lighting, had free access to food and water, and were used irrespective of gender. Animal protocols compliant with §4 of the German law of animal protection were reviewed and approved by the Tübingen University committee on animal protection (Einrichtung für Tierschutz, Tierärztlichen Dienst und Labortierkunde; Registration No.: 08/12/2015, 22/05/2014) and by the Service de la consommation et des affaires vétérinaires du Canton de Vaud (VD1367.5). All the experiments were performed in accordance with the ARVO statement for the use of animals in ophthalmic and visual research.

### Retinal Explant Cultures

Retinal explant cultures were prepared as previously published^42^. Briefly, the eyes were enucleated and incubated for 15 min at 37 °C in pre-warmed 0.12% proteinase K (Sigma-Aldrich, Hamburg, Germany; P-6556) in basal R16 medium (Thermo Scientific, Rockfort, Illinois, USA; 07490743 A). To stop enzymatic activity, eyes were rinsed in basal R16 medium with 10% Sera Plus Fetal Calf Serum (FCS; PAN Biotech GmbH, Aidenbach, Germany; P30-3701) and then washed in serum-free basal medium. Cornea, lens, sclera, and choroid were removed carefully, with only the RPE remaining attached to the retina. Finally, the retina was cut at four sides so it could spread flat like a clover-leaf, with RPE facing the membrane of the cell culture insert (0.45 μm; Merck Millipore, Tullagreen, Ireland; PIHA03050). The culture medium was changed every other day during 6, 12, or 19 culturing days. Retinal explants were left without treatment for two days (until P7), followed by olaparib treatment (10 nM to 50 μM; Biomol, Hamburg, Germany; BPS-27003). Olaparib was prepared in dimethyl sulfoxide (DMSO; Sigma-Aldrich, Hamburg, Germany; D-8779) and diluted in R16 serum free culture medium with supplements. For controls, the same amount of DMSO was diluted in culture medium.

### Assessing the intravitreal clearance of olaparib

The intravitreal clearance (CL_ivt_) of olaparib was calculated *in silico* using the QSPR model^27^. The chemical structure of olaparib was retrieved from ACD/Dictionary from ACDlabs software (version 12, Advanced Chemistry Development, Inc., Toronto, Canada) and 30 molecular descriptors were generated: pK_a_ for the most acidic molecular form, pK_a_ for the most basic form, LogD at pH 5.5 and 7.4, LogP, MW, PSA (polar surface area), FRB (freely rotatable bonds), HD (hydrogen bond donors), HA (hydrogen bond acceptors), Htot (HD + HA), rule of 5, molar refractivity, molar volume, parachor, index of refraction, surface tension, density, polarizability, C ratio, N ratio, NO ratio, hetero ratio, halogen ratio, number of rings and number of aromatic, 3-, 4-, 5- and 6-membered rings. The PCA score plot of the training set of the model including olaparib was inspected (Supplementary Fig. 4). Olaparib was found to be within the applicability domain of the model and thus predictable by the model. The intravitreal clearance value of olaparib was then calculated using the QSPR model: LogCL_ivt _= −0.25269–0.53747 (LogHD) + 0.05189 (LogD_7.4_), with the corresponding values of HD and LogD_7.4_ of olaparib. Half-life was calculated using equation t_1/2_ = ln2 V_d_/CL, where V_d_ is the volume of distribution and CL is the intravitreal clearance.

The half-life obtained in rabbit eyes was scaled down to mice eyes using the following rationale: Small lipophilic compounds are cleared from the vitreous mainly through the RPE^43^. The CL_ivt_ of small lipophilic compounds in mice is expected to be 30 times smaller than in rabbits. This is based on the equation CL = P × S, where P is the drug permeability in the RPE and S is the surface area of the RPE. The RPE surface areas in mice and rabbits are 16.5 mm^2 ^44 and 520 mm^2^ ; ref. 45, respectively. Assuming similar permeability of mouse and rabbit RPE, the intravitreal clearance in mouse would be 0.021 ml/h (rabbit value: 0.665 ml/h). The expected half-life in the vitreous can thus be calculated using the equation: t_1/2_ = ln2 Vd/CL (Vd = anatomical volume of the vitreous; in mouse 5 μl) giving an expected half-life of olaparib in the mouse vitreous of about eight minutes.

### *In vivo* treatment on rd1 animals

Mice were anesthetized by an intraperitoneal injection of ketamine (100 mg/kg) and xylazine (5 mg/kg), their pupils dilated with tropicamide eye drops (Mydriaticum Stulln; Pharma Stulln GmbH, Germany), and eye lids anesthetized locally with Novesin (Omnivision, Puchheim, Germany). Olaparib was diluted in DMSO and single intravitreal injections were performed at P11 on one eye. As contralateral control, the other eye was sham injected with the same concentration of DMSO (0.0003%) in sterile PBS. The *in vitro* data suggested 0.1 μM olaparib as the most effective concentration. To obtain this concentration *in vivo*, intravitreal injections were performed with 0.5 μl of a 1 μM olaparib solution. Assuming an average mouse eye volume of 5 μl and even compound distribution, this gave an effective intraocular concentration of 0.1 μM olaparib. Twelve *rd1* mice were used for intravitreal injections, six of these animals were sacrificed two days after treatment (P13), while the other six were sacrificed at P15. The eyes were enucleated immediately, fixed and prepared for cryosectioning.

### Fixation and sectioning

Eyes obtained from the *in vivo* study were fixed in 4% paraformaldehyde (PFA) for 75 min, while explant cultures were fixed for 45 min. After fixation, tissues were washed for 10 min in PBS. For cryoprotection, they were incubated in 10% sucrose solution for 10 min, 20% sucrose solution for 20 min, and 30% sucrose solution for at least 30 min. The retinas were frozen in Tissue-Tek O.C.T. Compound (Sakura Finetek Europe, Alphen aan den Rijn, Netherlands; 4583)-filled boxes. 12 μm tissue sections were prepared on a Leica CM3050S Microtome (Leica Biosystems, Wetzlar, Germany), thaw-mounted onto Superfrost Plus Object slides (R. Langenbrinck, Emmendingen, Germany; 03–0060).

### TUNEL assay

The terminal deoxynucleotidyl transferase dUTP nick end labelling (TUNEL) assay was performed on cryosections from treated/untreated wt and *rd1* retinas, using an *in situ* cell death detection kit conjugated with fluorescein isothiocyanate (Roche Diagnostics, Mannheim, Germany; 11 684 795 910). The sections were mounted in Vectashield with 4’,6-diamidino-2-phenylindole (DAPI) as a nuclear counterstain (Vector Laboratories, Burlingame, California, USA; H-1200).

### PAR staining

3,3′-diaminobenzidine (DAB) staining was performed with quenching of endogenous peroxidase activity with 40% MeOH and 10% H_2_O_2_ in PBS for 20 min. The sections were incubated with 10% normal goat serum (NGS) in PBS containing 0.1% Triton X-100 for 1 h followed by anti-PAR antibody (1:200; Enzo Life Sciences, Lörrach, Germany; ALX-804-220-P100) incubation for 1 h. Incubation with the biotinylated secondary antibody (1:150, Vector Laboratories Inc., Burlingame, CA, USA; BA-9200; in 5% NGS in PBST) for 1 h was followed by application of Vector ABC-Kit (Vector Laboratories, Burlingame, California, USA; PK-4000) for 1 h. DAB staining solution (0.05 mg/ml NH_4_Cl, 200 mg/ml glucose, 0.8 mg/ml nickel ammonium sulphate, 1 mg/ml DAB, 0.1 vol. % glucose oxidase in PB) was applied evenly, incubated for exactly 60 s and immediately rinsed with PB to stop the reaction. The sections were mounted in Aquatex (Merck, Darmstadt, Germany; 1.08562.0050).

### Immunofluorescence staining

Tissue sections were blocked and permeabilised in 10% BSA and 10% normal serum in PBS containing 0.1% Triton X-100 and incubated overnight in primary antibody in blocking solution. Primary antibody sources and dilutions are listed in Table 1. To increase the visibility of cones, TN-XL biosensor was enhanced by staining against its EGFP domain. Secondary antibodies were anti-sheep and -rabbit IgG’s, respectively, coupled to Alexa488 (1:350; Life Technologies, Carlsbad, California, USA). The sections were mounted in Vectashield (Vector Laboratories) with DAPI to visualize cell nuclei. Serial sections processed similarly, but without primary antibody, were used to control for non-specific background.

### Microscopy and cell counting

The mounted cultures were analyzed using Zeiss Axio Imager Z1 ApoTome microscope, AxioCam MRm camera and Zeiss AxioVision 4.7 software in Z-stack (3 slices per picture; slice distance: 14 μm) and mosaic mode at 20 × magnification. For quantitative analysis, positive cells in the entire ONL of four cross-sections per culture were counted manually. The percentage of positive cells was calculated by dividing the absolute number of positive cells by the total number of ONL cells. which was assessed by dividing ONL area by the size of a photoreceptor nucleus (17.3 μm^2^), as measured via DAPI staining. Photoreceptor rows were assessed by counting the individual nuclei lining up in one ONL column, every 200 μm and averaging the counts. Cone density was calculated by counting GFP positive somata per 100 μm of ONL.

Graphs were prepared in GraphPad Prism 6 (GraphPad Software, La Jolla, CA, USA); Adobe Photoshop CS5, and Corel DRAW X3 were used for image processing.

### Western Blot

Retinal tissue from *wt* and *rd1* mice were homogenized in modified RIPA lysis buffer (50 mM trizma base, 150 mM NaCl, 19 mM Na_4_O_7_P_2_, 1 mM EDTA, 1 vol% Triton-X-100, 1 mM DTT and 0.1 vol% of protease inhibitor cocktail III EDTA-free (EMD Millipore Corp., Billerica, Massachusetts, USA); pH = 7.4) with a Precellys homogenisator (Bertin Technologies, Montigny le Bretonneux, France). For separation of proteins, 25 μg protein per well were loaded onto a 12% SDS-PAGE gradient gel, and run at 120 V. Subsequently, the proteins were transferred to PVDF membranes (Merck Millipore, Tullagreen, Ireland). Roti block buffer (Roth, Karlsruhe, Germany) was applied for 3 h at room temperature. Membranes were incubated in primary antibodies against PAR (1:1000; see above) or actin (1:400; Abcam, Milton, UK; Ab1801) in buffer containing PBST and 5% dried milk (Carl Roth GmbH, Karlsruhe, Germany) overnight at 4 °C. Membranes were washed with PBST and incubated with secondary antibodies labelled with IRDye680 RD (LI-COR Biotechnology GmbH, Bad Homburg, Germany; 926–68070) or IRDye800 CW (LI-COR Biotechnology GmbH, Bad Homburg, Germany; 926–32211) for 1 h at room temperature. LI-COR Odyssey Sa Infrared Imaging System (LI-COR Biotechnology GmbH, Bad Homburg, Germany) was used for detection of fluorescent protein bands, which were quantified using ImageJ (National Institute of Health, Washington, USA).

### Statistics

Statistical analysis was performed using GraphPad Prism 6 software and Kruskal-Wallis test for multiple analyses or two-sample Komolgorov-Smirnov test. For one-to-one group comparisons Student’s t-test as implemented in Microsoft Excel software (Microsoft Corporation, Seattle, WA, USA) was used.

## Additional Information

**How to cite this article**: Sahaboglu, A. *et al*. Olaparib significantly delays photoreceptor loss in a model for hereditary retinal degeneration. *Sci. Rep.* **6**, 39537; doi: 10.1038/srep39537 (2016).

**Publisher's note:** Springer Nature remains neutral with regard to jurisdictional claims in published maps and institutional affiliations.

## Supplementary Material

Supplementary Figures

## Figures and Tables

**Table 1:** Primary antibodies used in this study.

Antigen	Species	Dilution	Company	Article number
5mC	Sheep	1:200	Novus Biologicals, Littleton, Colorado, USA	NB100-744
5hmC	Rabbit	1:200	Active Motif, Carlsbad, California, USA	39770
cGMP	Sheep	1:500	*kindly provided by Jan de Vente, University of Maastricht, Netherlands*	
EGFP	Rabbit	1:500	Merck-Millipore, Darmstadt, Germany	AB2080
