# Supplementary material for: Olaparib significantly delays photoreceptor loss in a model for hereditary retinal degeneration
Source: Sci Rep. 2016 Dec 22;6:39537. doi: 10.1038/srep39537 (PMC5177898; doi:10.1038/srep39537)
Supplement: Supplementary Figures [file srep39537-s1.pdf]

Olaparib significantly delays photoreceptor loss in a model for hereditary retinal degeneration

Ayse Sahaboglu*[1], Melanie Barth*[1,2], Enver Secer[1,3], Eva M. del Amo[4], Arto Urtti[4,5], Yvan Arsenijevic[6], Eberhart Zrenner[1], François Paquet-Durand[1]

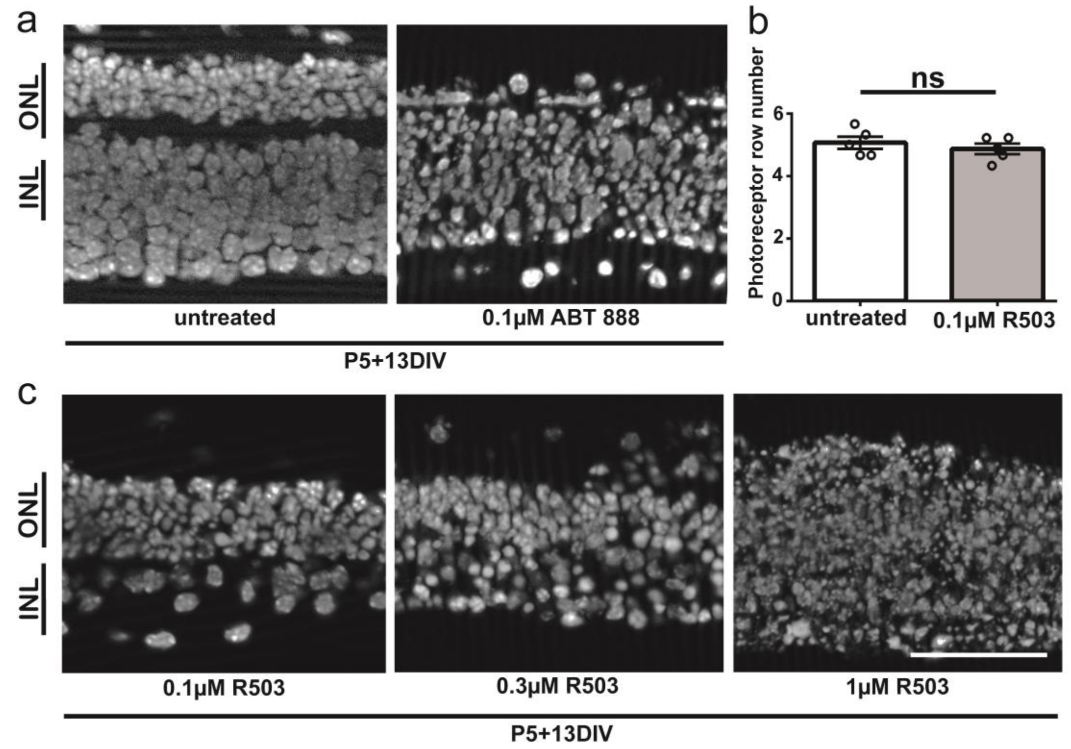

Figure S1 | Effect on photoreceptor viability and retinal morphology of two different PARP inhibitors. (A) At P18 DAPI staining for cell nuclei showed an unorganized retinal structure with no clear tissue layering in ABT-888 treated cultures compared to untreated. (B) No significant differences were found for the number of photoreceptor rows when 0.1 µM R503 cultures were compared to control. (C) Treatment with higher concentrations of R503 showed a toxic effect to retina. Bar graphs represent means ± SEM. n(*rd1*, untreated) = 6; n(*rd1*, 0.1 µM ABT-888) = 4; n(*rd1*, 0.1 µM R503) = 4; n(*rd1*, 0.3 µM R503) = 4; n(*rd1*, 1 µM R503) = 4.

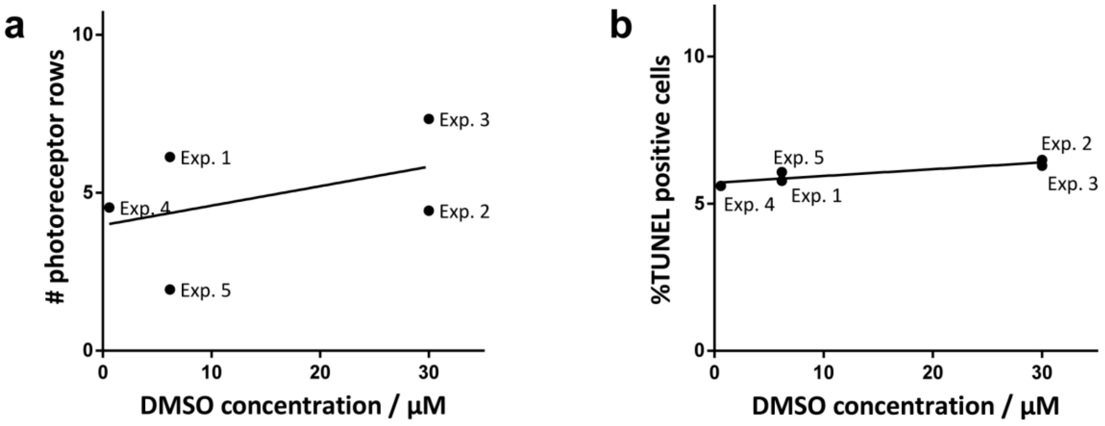

Figure S2 | DMSO did not significantly affect photoreceptor cell death and survival. (A) The number of photoreceptor rows (B) and the percentage of TUNEL positive cells were plotted against the respective DMSO concentration used in control retinal culture experiments. Exp. 1, 2… = first, second, *etc*. experiment.

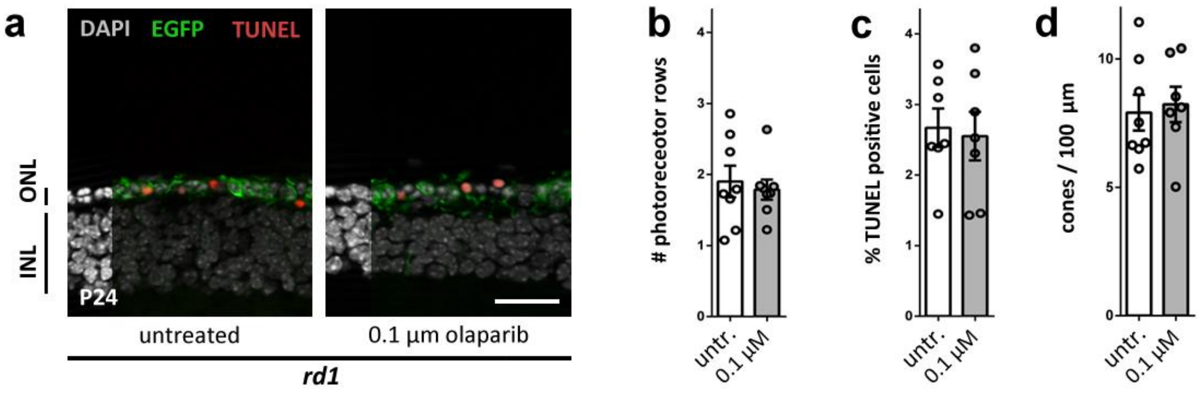

Figure S3 | Long-term effect of olaparib treatment to *rd1*$^{TN\text{-}XL}$ retinal cultures at P24. (A) Immunohistochemical staining showed no change in treated *vs*. untreated cultures at P24. (B) No significant differences were found for the number of photoreceptor rows, (C) the percentage of TUNEL positive cells, (D) or cone density. Bar graphs represent means ± SEM. n(*rd1*, untreated) = 8; n(*rd1*, 0.1 µM olaparib) = 7.

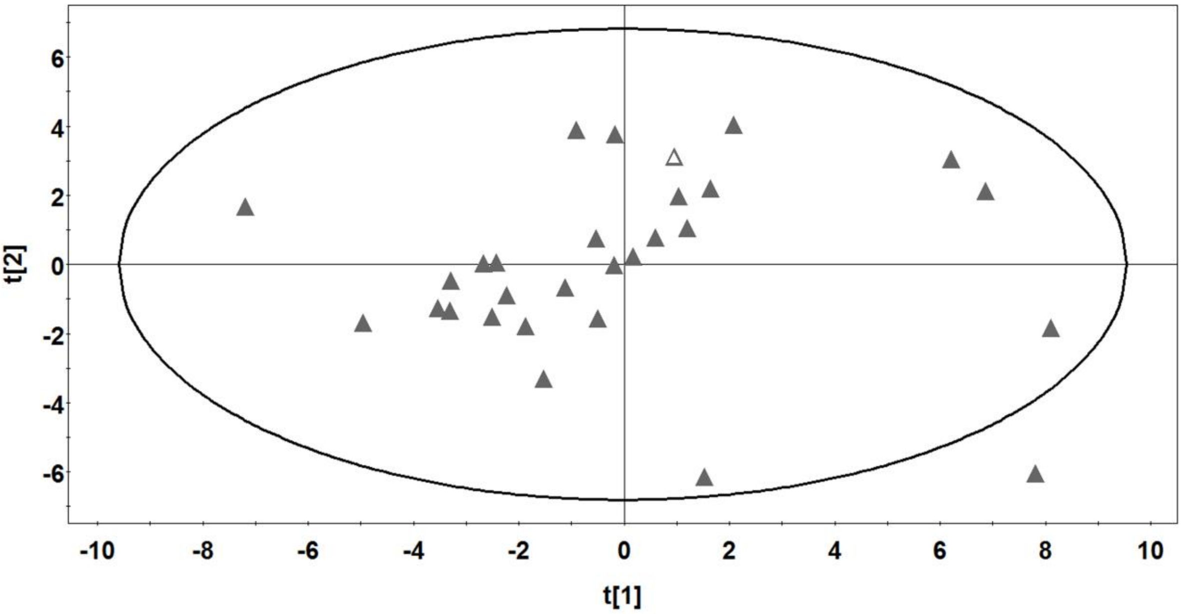

Figure S4 | The intravitreal clearance model is applicable to olaparib. The PCA score plot of the training set of the intravitreal clearance model [27]. The ellipse indicates the applicability domain of the model, with the full triangles representing the compounds used to build the model. Note that the single open triangle, corresponding to olaparib, falls inside the chemical space of the model. The intravitreal clearance of olaparib can thus be predicted by the model.

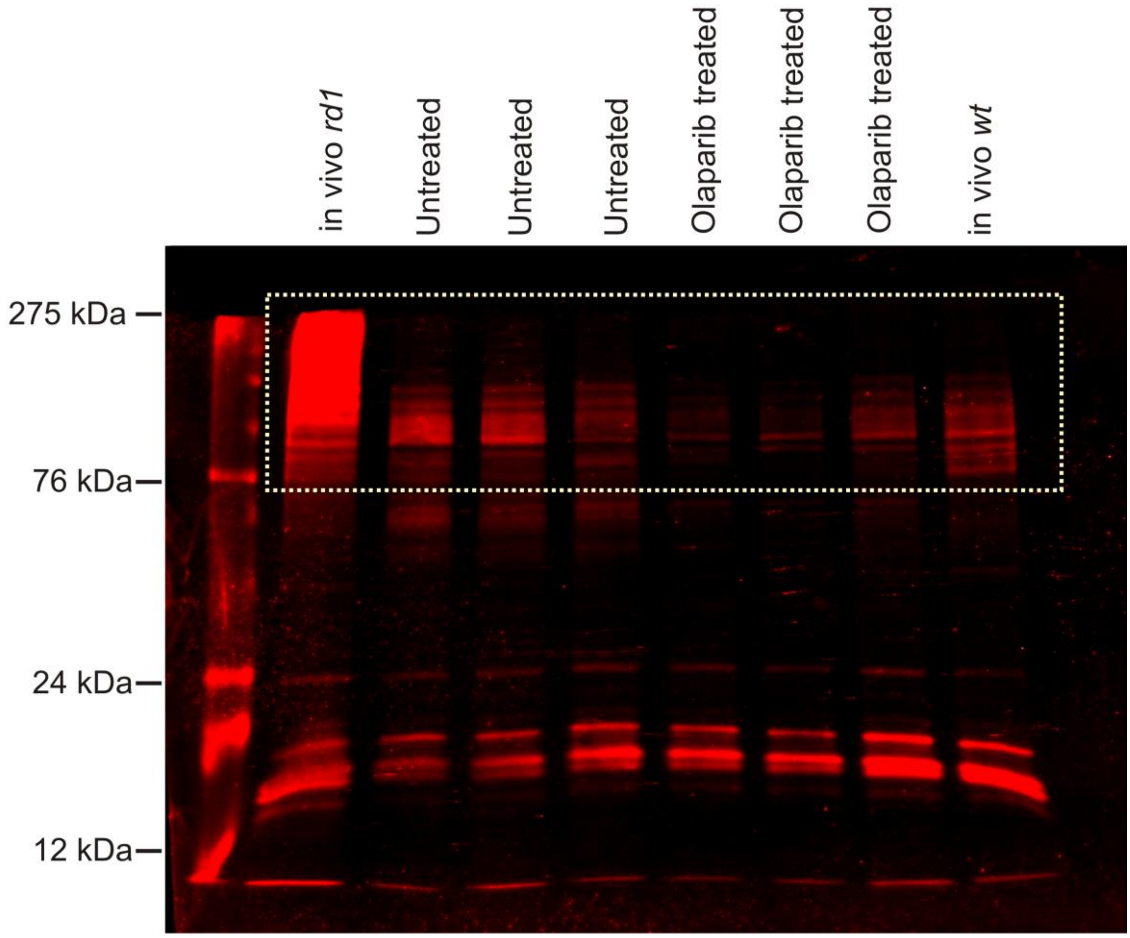

Figure S5 | Western blot analysis of PARylated proteins. Western blotting confirmed previous observations[8, 9] on the strong PARylation of proteins in P11 *rd1* retina *in vivo* (first sample lane) when compared to age-matched wildtype (wt; last lane). In P11 *rd1* retinal explant cultures *in vitro* PARylation was less prominent compared to the *in vivo* situation. Importantly, in *rd1* cultures treated with 100 nM olaparib there was a strong reduction of PARylation. The left-most lane shows the molecular weight standard; the area surrounded by the dashed white line was used for the quantification of PARylation (*cf*. Fig. 2e).
